# In-depth mining of single-cell transcriptome reveals the key immune-regulated loops in age-related macular degeneration

**DOI:** 10.3389/fnmol.2023.1173123

**Published:** 2023-05-19

**Authors:** Wencan Wang, Peng Lin, Siyu Wang, Guosi Zhang, Chong Chen, Xiaoyan Lu, Youyuan Zhuang, Jianzhong Su, Hong Wang, Liangde Xu

**Affiliations:** ^1^National Engineering Research Center of Ophthalmology and Optometry, Eye Hospital, Wenzhou Medical University, Wenzhou, China; ^2^State Key Laboratory of Ophthalmology, Optometry and Visual Science, Eye Hospital, Wenzhou Medical University, Wenzhou, China; ^3^National Clinical Research Center for Ocular Diseases, Eye Hospital, Wenzhou Medical University, Wenzhou, China; ^4^Institute of PSI Genomics Co., Ltd., Wenzhou, China; ^5^Center of Optometry International Innovation of Wenzhou, Eye Valley, Wenzhou, China

**Keywords:** age-related macular degeneration (AMD), single cell transcriptome sequencing (scRNA-seq), cell communication, regulon, regulation loops

## Abstract

**Introduction:**

Age-related macular degeneration (AMD), an ever-increasing ocular disease, has become one of the leading causes of irreversible blindness. Recent advances in single-cell genomics are improving our understanding of the molecular mechanisms of AMD. However, the pathophysiology of this multifactorial disease is complicated and still an ongoing challenge. To better understand disease pathogenesis and identify effective targets, we conducted an in-depth analysis of the single-cell transcriptome of AMD.

**Methods:**

The cell expression specificity of the gene (CESG) was selected as an index to identify the novel cell markers. A computational framework was designed to explore the cell-specific TF regulatory loops, containing the interaction of gene pattern signatures, transcription factors regulons, and differentially expressed genes.

**Results:**

Three potential novel cell markers were *DNASE1L3* for endothelial cells, *ABCB5* for melanocytes, and *SLC39A12* for RPE cells detected. We observed a notable change in the cell abundance and crosstalk of fibroblasts cells, melanocytes, schwann cells, and T/NK cells between AMD and controls, representing a complex cellular ecosystem in disease status. Finally, we identified six cell type related and three disease-associated ternary loops and elaborated on the robust association between key immune-pathway and AMD.

**Discussion:**

In conclusion, this study facilitates the optimization of screening for AMD-related receptor ligand pathways and proposes to further improve the interpretability of disease associations from single-cell data. It illuminated that immune-related regulation paths could be used as potential diagnostic markers for AMD, and in the future, also as therapeutic targets, providing insights into AMD diagnosis and potential interventions.

## 1. Introduction

Age-related macular degeneration (AMD) is one of the most critical eye diseases that cause age-related blindness. It is estimated that, by 2040, the number of people with AMD globally is expected to be ~300 million, thus posing a significant public health problem with substantial socioeconomic implications (Wong et al., 2014). AMD is a multifactorial disease. Previous reports inferred that genetic factors, environmental influences, congenital disabilities, nutritional disorders, inflammation, metabolic disorders, and other factors may be involved in AMD progression (Schmidl et al., 2015; Mitchell et al., 2018; Jin et al., 2019; Fleckenstein et al., 2021; Guymer and Campbell, 2023). However, until now, the specific expression characteristics and pathogenesis of macular degeneration have not been fully studied, and it is still a great challenge to further understand the pathogenesis of AMD.

At present, prophylactic therapies have little effect, and the treatment strategies mainly focus on the restraint of neovascularization (Chakravarthy and Peto, 2020). Although currently some medicines (laser thermal photocoagulation, photodynamic therapy with verteporfin, and drug therapy with anti-vascular endothelial growth factor) may slow the progression of vision loss, or improve vision in some cases, none of these therapies prevent the recurrence of neovascularization and each of these must be reapplied to prevent the symptom from worsening (Stahl, 2020). Moreover, clinical data show that there are extensive individual differences in the effect of anti-VEGF therapy, and the underlying reason is still unclear (Thomas et al., 2021). Therefore, developing individual differential diagnoses and effective precise therapies for AMD is imperative. Recently, the development of single-cell RNA sequencing (scRNA-seq) provided the opportunity for high-throughput sequencing of the transcriptome at the single-cell level, which can investigate gene expression within a single cell while solving the complex problems of cell heterogeneity. It is possible to parse individual cells' behavior, mechanisms, and relationships to each other.

For now, scRNA-seq has been widely used in various disease research fields, including eye diseases such as AMD, retinal degeneration, and ocular tumors. For instance, Lehmann et al. revealed the cellular and molecular landscape of adult RPE/choroid and uncovered a Hedgehog-regulated choroidal immunomodulatory signaling circuit, opening up a new way to study retinal vascular diseases and choroidal-associated inflammatory disorders causing blindness (Lehmann et al., 2020). Voigt et al. explored the molecular mechanism of choroid vascular disease and its influence on AMD, providing potential ideas for treating it (Voigt et al., 2019). Menon et al. performed massively parallel single-cell RNA sequencing of human retinas, and GWAS-based enrichment analysis identified glia, vascular cells, and cone photoreceptors associated with AMD risk (Menon et al., 2019). Although previous studies have explained some molecular mechanisms of AMD, the specific molecular regulatory processes in AMD still require further exploration.

In the present study, six single-cell RNA-sequencing samples from the macula and periphery of three human donor eyes were selected, and in-depth analysis was conducted to explore the novel potential markers, change in cell–cell crosstalk, unique gene expression patterns, and transcription factor regulons of the primary cell types in AMD patients and controls.

## 2. Methods

### 2.1. Data collection

Processed single-cell transcriptomic datasets of three donors, including two controls (donors 1 and 2) and one AMD (donor 3), were obtained from the Gene Expression Omnibus (GEO, https://www.ncbi.nlm.nih.gov/geo/) database with the following accession numbers: GSE135922. The single-cell transcriptomic data of the macula and periphery were obtained for each donor. Two external datasets were used as validation data: external dataset 1. Eye data of Tabula Sapiens Consortium study (Tabula Sapiens et al., 2022), including 10,650 cells from 3 donors, external dataset 2. GSE188280 (Supplementary Table S1), including 8 samples taken from two early-stage AMD patients with macula choroid, macula retina, peripheral choroid, and peripheral retina. The analysis steps are consistent across the three data sets.

### 2.2. Clustering of the single-cell data matrix

The clustering analysis was performed by the R package “Seurat” (Hao et al., 2021). The “*SCTransform*” function was selected to remove the inherent variation caused by mitochondrial gene expression. The highly variable genes were identified with the “*FindVariableFeatures*” function. Genes with higher variation were used to perform principle component analysis (PCA). The '*ElbowPlot*' function determined the number of significant principle components (PCs). The k-nearest neighbor algorithm-based clustering was then performed to generate cell clusters, using the “*FindNeighbors*” and “*FindClusters*” functions with optimized “resolution” values for analysis. Finally, the clustering results were visualized using Uniform Manifold Approximation and Projection (UMAP).

### 2.3. Differential expression analysis

The differentially expressed genes (DEGs) were calculated using the R package “Seurat” “*FindAllMarkers*” function (Hao et al., 2021). We separately analyzed the heterogeneity of expression between various cells and between AMD and control samples. Statistical significance was determined using an adjusted *p-*value (*p_val_adj*) of < 0.05 and the Seurat default threshold of average log2 fold change (avg_log2FC) ≥0.25.

### 2.4. Cell type annotation and enrichment

Major cell clusters were manually annotated based on the cell-specific markers obtained from the CellMarker database (Zhang et al., 2019) and publicly published literature. The abundance of cell types (*ACTs*) across different conditions (i.e., AMD, control, macula, and periphery) was quantified to adjust the influence of the differences in samples. It assumes that one cell type is enriched under a specific condition *k* if *ACT* > 1 (Zhang et al., 2021).

### 2.5. Novel cell marker identification

The expression of genes with an avg_log2FC larger than 1 and *p_val_adj* < 0.05 was reserved for further study. The cell expression specificity of the gene (*CESG*) was selected as an index to identify the novel cell markers and calculated as follows:


$$\begin{array}{l} CESG_{i}=\frac{{pct.1}_{i}}{{pct.2}_{i}}=\frac{\frac{m_{ij}}{M_{j}}}{\frac{K_{i-j}}{\left(N-M_{j}\right)}} \end{array}$$

where *pct*.1_*i*_ and *pct*.2_*i*_ represent the percentage of cells expressing *i* gene in one cell type and the others, respectively. *m*_*ij*_ is the number of expressing *i* gene in *j* cell type. *M*_*j*_ is the total number of cells in *j* cell type. *K*_*i*−*j*_ represents the number of expressing *i* gene in other cell types except *j* cells. *N* is the total number of cells in all cell types. The gene with *CESG* greater than the known markers of the cell type is recognized as the candidate cell marker. After literature mining, the candidate novel cell markers are regarded as novel cell markers if there are no records in the previous reports.

### 2.6. Cell–cell communication analysis

The R package “Cellchat” (Jin et al., 2021) was used to analyze crosstalk among all cell types. The interactions between gene expression, signaling from ligands and receptors, and their cofactors were integrated to establish the probability of cell–cell communication. The “*computeCommunProb*” and “*filterCommunication*” functions were used to infer the communication network at the ligand–receptor level. The cellular communication network at the signaling pathway level was established by the “*computeCommunProbPathway*” function.

### 2.7. Gene expression pattern identification

We identified cell type-specific gene expression patterns by the R package “scCoGAPS” (Stein-O'brien et al., 2019). Using the non-negative matrix factorization (NMF) algorithm, scCoGAPS decomposes a matrix M of G genes (rows) and S samples (columns) into two matrices containing gene weights, the amplitude (A?R^G × *k*^) matrix, and sample weights, the pattern (P?R^k × *S*^) matrix. The predictive power was calculated to assess the relationship between the pattern and each cell type using “ProjectR” (Sharma et al., 2020) and the “*aucMat*” function. The predictive power ranged from 0 to 1, and the closer the value is to 1, the stronger predictive power of the pattern for each cell type annotation. The “*pheatmap*” function was used to visualize blue-red scale heatmaps.

### 2.8. Transcription factor regulon identification

Transcription factor regulons were inferred using the R package “SCENIC” (Aibar et al., 2017). The function “*runGenie3*” was used to calculate the weight of correlation between each TF and gene, and the function '*runSCENIC_1_coexNetwork2modules*' was used to identify gene sets that were co-expressed with TF. “*runSCENIC_2_createRegulons*” function was used to prune the obtained co-expression modules based on the cisTarget database (Imrichova et al., 2015) (https://resources.aertslab.org/cistarget/). Only genes with significant enrichment of the regulator's binding motif are retained. Finally, we used the “*runSCENIC_3_scoreCells*” function to calculate regulon activity scores (*RAS*) in each cell with AUCell, and the regulon specificity score (*RSS*) was calculated by the “*calcRSS*” function to evaluate the activities associated with each cell type (Suo et al., 2018). The *RSS* is defined by converting Jensen–Shannon divergence (*JSD*) to a similarity score:


$$\begin{array}{l} RSS\left(R,C\right)=1-\sqrt{JSD\left(P^{R},P^{C}\right)} \end{array}$$

where the vector $P^{R}=\left(p_{1}^{R},\ldots ,p_{n}^{R}\right)$ represents the distribution of RAS in the cell population (*n* is the total number of cells). Here, the RAS are normalized so that $\overset{n}{\underset{i}{\sum }}p_{i}^{R}=1$. The vector $P^{C}=\left(p_{1}^{C},\ldots ,p_{n}^{C}\right)$ indicates whether a cell belongs to a specific cell type ($p_{i}^{C}=1$) or not ($p_{i}^{C}=0$). This vector is also normalized so that $\overset{n}{\underset{i}{\sum }}p_{i}^{C}=\;1$.

### 2.9. Cell-specific TF regulatory loop construction

We defined *P*_*loop*_ by considering the principle of the hypergeometric algorithm to quantitatively evaluate the regulatory relationship between gene expression pattern, regulon, and DEGs in AMD progression. Here, we only focused on the gene expression patterns with high predictive power (≥ 0.7), regulons with significantly specific *RSS* (*RSS* ≥ 0.1 and normalization of *RAS* (Z score) ≥ 2 in all cell types or *RSS* with |log2FC|>1 between AMD and controls), and DEGs (*p_val_adj* < 0.05) in each cell type without and with disease properties. In each of the three types of gene sets, we calculated *P*_*loop*_ between pairs (gene expression pattern, regulon, and DEGs) separately. Only pairs with *P*_*loop*_ < 0.05 were selected to construct the cell-specific TF regulatory loops.


$$\begin{array}{l} P_{loop}=\frac{C_{M}k*{∁}_{N-M}^{n-k}}{{∁}_{N}^{n}} \end{array}$$

where *k* is the number of intersecting genes between node_1 (i.e., a gene expression pattern, a regulon, or DEGs) and node_2 (i.e., a gene expression pattern, a regulon, or DEGs), where node_1 is different from node_2. *N* is the total number of genes in single-cell transcriptomic datasets (used in the present analysis). *M and n* are the numbers of genes in node_1 and node_2, respectively.

## 3. Results

### 3.1. Identification of cell types and novel cell markers

To explore the heterogeneity, cellular diversity, and transcriptome changes between patients with AMD and controls, we performed an in-depth analysis of single-cell transcriptomic data. A summary of three donors is shown in Supplementary Table S2. Cells from RPE/choroid regions were extracted for single-cell RNA sequencing (scRNA-seq), including the macula and periphery. In total, 4,335 cells (2,167 cells from the macula and 2,168 cells from the periphery) and 21,040 genes were detected. The first 15 PCs were used to cluster cells with similar gene expression profiles (Supplementary Figure S1). Finally, 12 distinct clusters were identified, ranging in size from 40 cells to 1,245 cells. Through principal component analysis of cell types (Supplementary Figure S2), we found that cell types for re-annotation were well-classified and delimited. All clusters were composed of cells from each donor, except for cluster 11, the smallest cluster, which did not contain any cells from donor 2 (Supplementary Table S3).

**Figure 1:**
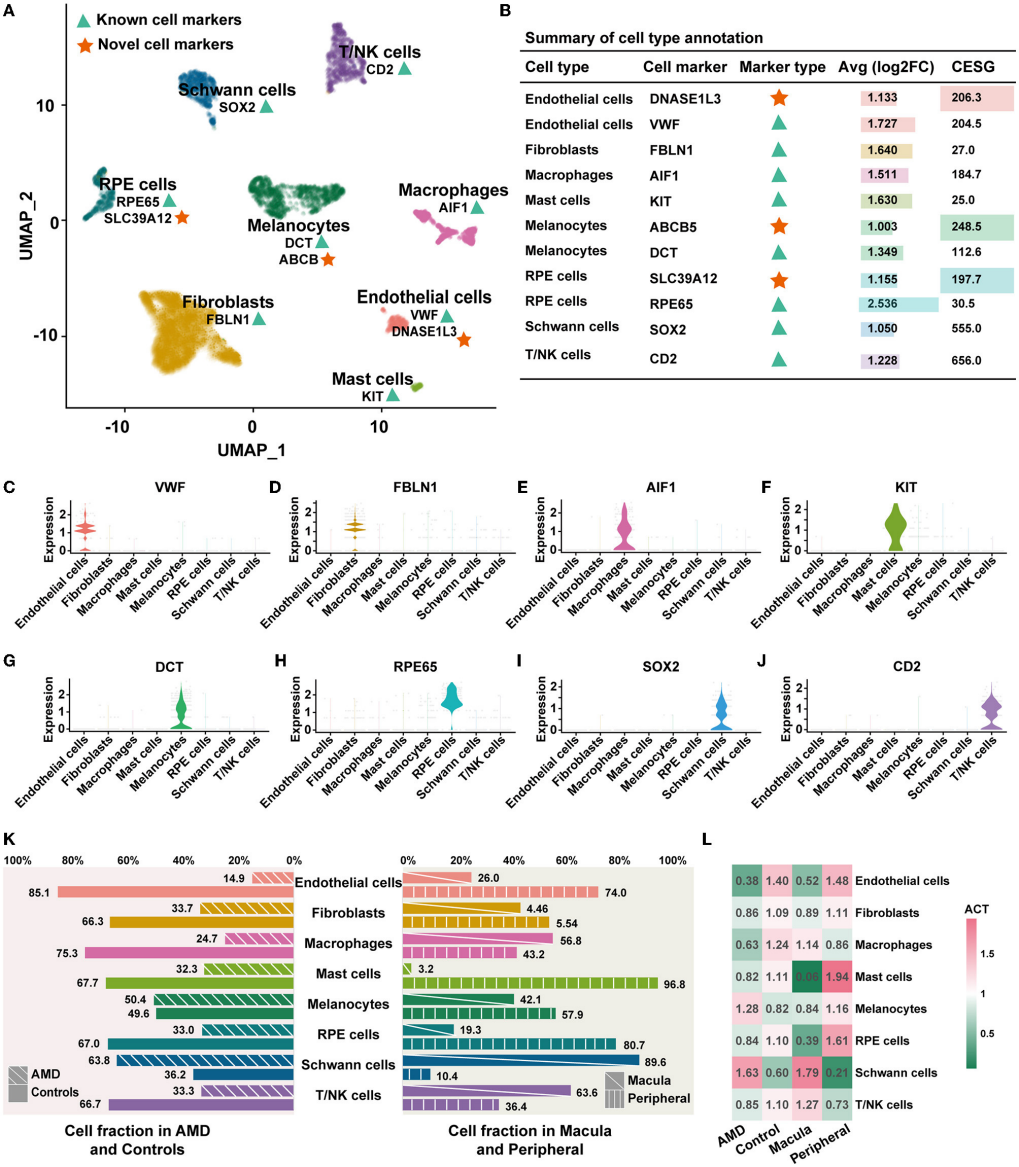
Cell composition and distribution. **(A)** UMAP dimensionality reduction for the 4,335 cells with eight cell types. The green triangles represent known cell markers, and the red stars represent the novel cell markers. **(B)** Summary of the cell type annotation. “Inf” indicates that the gene is only specifically expressed in one cell type and is not expressed in the other cell types. **(C–J)** Violin plots illustrating the identity of each cluster through well-known cell type-specific markers. **(K)** Cell abundant in AMD, control, macula, and peripheral. **(L)** ACT of each cell type.

Annotation of cell types was based on the published cell markers and specifically highly expressed genes of each cluster (Figure 1A). Detailed markers used for cell types are shown in Figure 1B (labeled with “known”) and visualized over the violin plot in Figures 1C–J. The 12 distinct clusters were interpreted as eight cell types, including endothelial cells, fibroblasts, macrophages, mast cells, melanocytes, RPE cells, Schwann cells, and T/NK cells. For each cluster, the reproducibility of gene expression was validated in an external dataset 1 (Supplementary Figure S3A). We observed that the top five highly expressed genes in each detected cluster showed 75% (9/12 clusters) expression identity in the external dataset 1. Two clusters (2/12) failed to repeat because of the absence of Schwann cells in external dataset 1. It largely corroborates the results of our cell type annotation.

In addition, we identified three novel potential cell-specific markers by enrolling the score of *CESG* (Figure 1B, details shown in Methods). Compared to the known marker above, all novel markers were differentially expressed with a higher specificity (indicated as a higher *CESG* and an average log2FC > 1). This specific expression trend was completely repeated in external dataset 1 (Supplementary Figure S3B). There are two novel potential markers in endothelial cells. *DNASE1L3* encodes a member of the deoxyribonuclease I family and acts in internucleosomal DNA fragmentation (INDF) during apoptosis and necrosis (Mizuta et al., 2013). In addition, *ABCB5* is the novel potential marker of melanocytes, which could promote melanoma metastasis by activating the NF-?B signaling pathway (Wang et al., 2017). *SLC39A12* is regarded as the novel potential marker of RPE cells. It is involved in protein, nucleic acid, carbohydrate, and lipid metabolism, as well as in the control of gene transcription, growth, development, and differentiation (Taylor, 2023).

### 3.2. Heterogeneity of cell types

Next, we counted the proportion and abundance of each cell type. We observed a significant difference in the cell fraction between the patients with AMD and controls, as well as in the macular and peripheral regions (Figure 1K). The populations of Schwann cells (337/191) and melanocytes (437/430) in the AMD patients were greater than those in the controls. To remove the effects of influencing factors from the uneven sampling of tissue or un-clarity number of cells, we calculated the abundance of cell types (*ACT*, details in Methods). The abundance of Schwann cells in AMD patients (*ACT* = 1.63) was 2.7 times larger than that of controls (*ACT* = 0.60), and in the macula (*ACT* = 1.79) was 8.5 times greater than that of peripheral (*ACT* = 0.21). In contrast, the abundance of endothelial cells, fibroblasts, macrophages, mast cells, RPE cells, and T/NK cell populations decreased AMD patients (Figure 1L). These observations indicate AMD patients with cellular heterogeneity and diversity. It may be one of the influencing factors in AMD onset and worsening. Decreasing key cells could alter the relevant signal transduction, leading to an imbalance in the cellular ecosystem.

### 3.3. Cell-to-cell Communications

**Figure 2:**
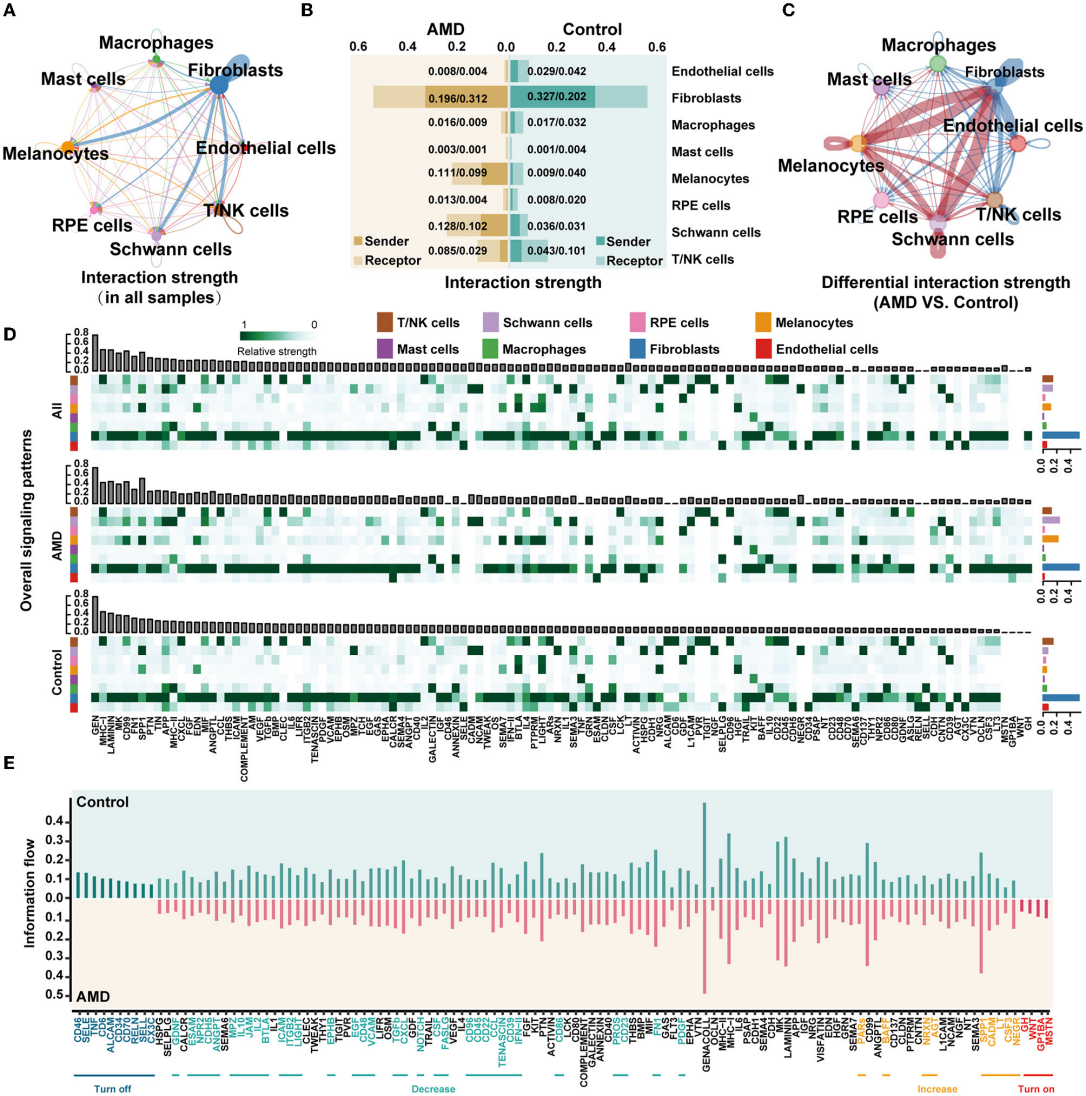
scRNA-seq presents the obvious changes in cell communication during AMD progression. **(A)** The interaction strength between cells in all samples. The thicker link indicates stronger strength. **(B)** The intensity of the signals sent and received by each cell in AMD and control. The horizontal axis indicates the interaction strength. **(C)** The difference in the interaction strength of cells between AMD and control. The red links indicate the interaction strength in AMD is stronger than that in control, while the blue lines are the opposite. **(D)** The overall signaling patterns in all samples, AMD, and control. The bar graph above shows the total strength of each signal of all cells, and the bar graph on the right shows the total strength of each cell of all signals. The darker the color of the heat map, the stronger the signal strength. **(E)** The information flow in AMD and control. Turn off represents the closed path in AMD, “Decrease” represents the reduced path in AMD, “Increase” represents the increased path in AMD, and “Turn on” represents the open path only in AMD.

To further understand how cells in patients cooperate and contribute to AMD pathogenesis, we detected communication between cells via ligand and receptor interactions. Figure 2A illustrates the integrated cell–cell communication network, illustrating the interaction strength of the eight primary cell types. We found that the strongest interactions occurred in fibroblasts. Then, statistics on the signal strength of each ligand–receptor pathway in each cell type also presented that fibroblasts were involved in most of the pathways with the strongest signals, followed by melanocytes, Schwann cells, and T/NK cells (the top sub-plot in Figure 2D and Supplementary Figure S4).

To further determine whether the change existed in cellular crosstalk between AMD patients and controls, we calculated the strength of signals sent and received by each cell type in the two groups of samples (Figure 2B), and further compared the cell communication under different disease states (Figure 2C). The red links between cell types represent the enhanced communication in the AMD group, while the blue links were the decreased signals. It is worth noting that the most significant changes were observed in fibroblast cells, followed by melanocyte and Schwann cells. In AMD patients, the interactions of fibroblasts with melanocytes and Schwann significantly enhanced, while the interactions of fibroblasts with the other cells weakened (Figure 2C). We suspect that the changed signals may associate with the increased counts of melanocytes and Schwann cells in AMD patients compared with controls (Figure 1K and Supplementary Table S4). In addition, we found that the signal strength of the ligand–receptor pathway in melanocyte and Schwann cells decreased in controls (Figure 2D, bottom, Supplementary Table S4). We inferred that the number of melanocyte and Schwann cells is reduced, weakening the relevant signals, which may be one of the factors leading to an imbalance in the immune response in the fundus area, but the detailed relationship needs more biological evidence to further quantify.

To further explore signal changes in the ligand–receptor pathway in AMD and controls, we calculated the relative information flow of signaling pathways, which was defined by the overall communication probability among cell types in each disease status (two bottom plots in Figure 2D). Figure 2E shows that 52.5% (63/120) pathways, such as ANNEXIN, EPHA, HSPG, SEMA3, and TWEAK maintain a similar flow between AMD and controls (black label in the horizontal axis). While the prominently changed pathways (47.5% (57/120), Wilcoxon test *p* < 0.05) were defined as four types according to the direction and the extent of information gain: (i) turn off (10 pathways, namely, ALCAM, CD6, CD34, CD46, CD70, CX3C, RELN, SELL, SELE, and TNF), (ii) turn on (four pathways, namely, GH, GP1BA, MSTN, WNT), (iii) decrease (34 pathways, including CD22, IL2, IL10, and TGFb), and (iv) increase (nine pathways, namely, AGT, BAFF, CADM, CSF3, LT, NEGR, NRXN, PARs, and SPP1). We did the same analysis in external dataset 2 and showed some repetition (Supplementary Figure S5). Although there were some differences, these differences might be due to differences between early and late AMD. Then, the trends in the changes in the four groups of information flows were also been validated by external dataset 2. The finding of the repeated tests indicated that 78.6% (11/14) of the specific change pathways in “turn off” and “turn on” were validated (Supplementary Figure S6). These results also suggest that some signals, such as TNF and CD46, change dynamically as the disease progresses from early to late stages. Meanwhile, functional of the remarkably altered pathway involved in a series of critical biological functions: immunity and inflammatory processes (e.g., ALCAM, CD6, CD34, CD46, CD70, CX3C, IL2, and IL10, *etc*.), nervous system (e.g., CSF, EPHB, GDNF, MSTN, NEGR, NOTCH, and NRXN, *etc*.), angiogenesis (e.g., EGF, PDGF, PROS, SPP1, TGFb, *etc*.), and cell survival (e.g., CXCL, PDGF, SPP1, SELE, and WNT, *etc*.).

Moreover, the non-negative matrix factorization was applied to identify the collaborative communication patterns of different cell types and their related key signal pathways. The communication patterns revealed four outgoing signaling flows and three incoming signaling flows in AMD (Supplementary Figures S7A, B), compared to six outgoing signaling flows and two incoming signaling flows in controls (Supplementary Figures S7C, D). It is further demonstrated that there is a clear difference in cell communication between AMD patients and controls. The output of AMD reveals that a large portion of outgoing fibroblast signaling is characterized by Pattern #1, which represents multiple pathways, including but not limited to AGT, BAFF, CSF3, CXCL, GH, IL1, LT, MK, MSTN, PDGF, and WNT (Supplementary Figure S7A). All of the outgoing endothelial cells, macrophages, melanocytes, and RPE cell signaling were characterized by Pattern #4, representing pathways such as BTLA, CD137, GRN, HGF, SEMA7, and SPP1. In contrast, the incoming communication patterns of AMD (Supplementary Figure S7B) show that endothelial cells, fibroblasts, and RPE cells were dominated by Pattern #1, which includes signaling pathways such as AGT, BAFF, CSF3, GH, GP1BA, LT, MSNT, NRXN, PARs, SPP1, and WNT, as well as CD46, CDH5, EGF, IFN–II, JAM, LIGHT, PDGF, TGFb, and VCAM among others. Macrophages, mast cells, and T/NK cell signaling were characterized by Pattern #3, driven by CCL, CD22, CD96, CXCL, IL2, and IL10 pathways. All the above results show that: (1) distinct cell types can rely on largely overlapping signaling networks; (2) one certain cell type, it activates different counts of signaling pathways, and the number of its involvement in cell communication and signaling pathways may be related to disease state; and (3) the same cell types in diverse disease states with different communication patterns and signaling pathways, which forms a specific cellular environment and participates in the biology process.

### 3.4. Differentially expressed genes between AMD and controls

**Figure 3:**
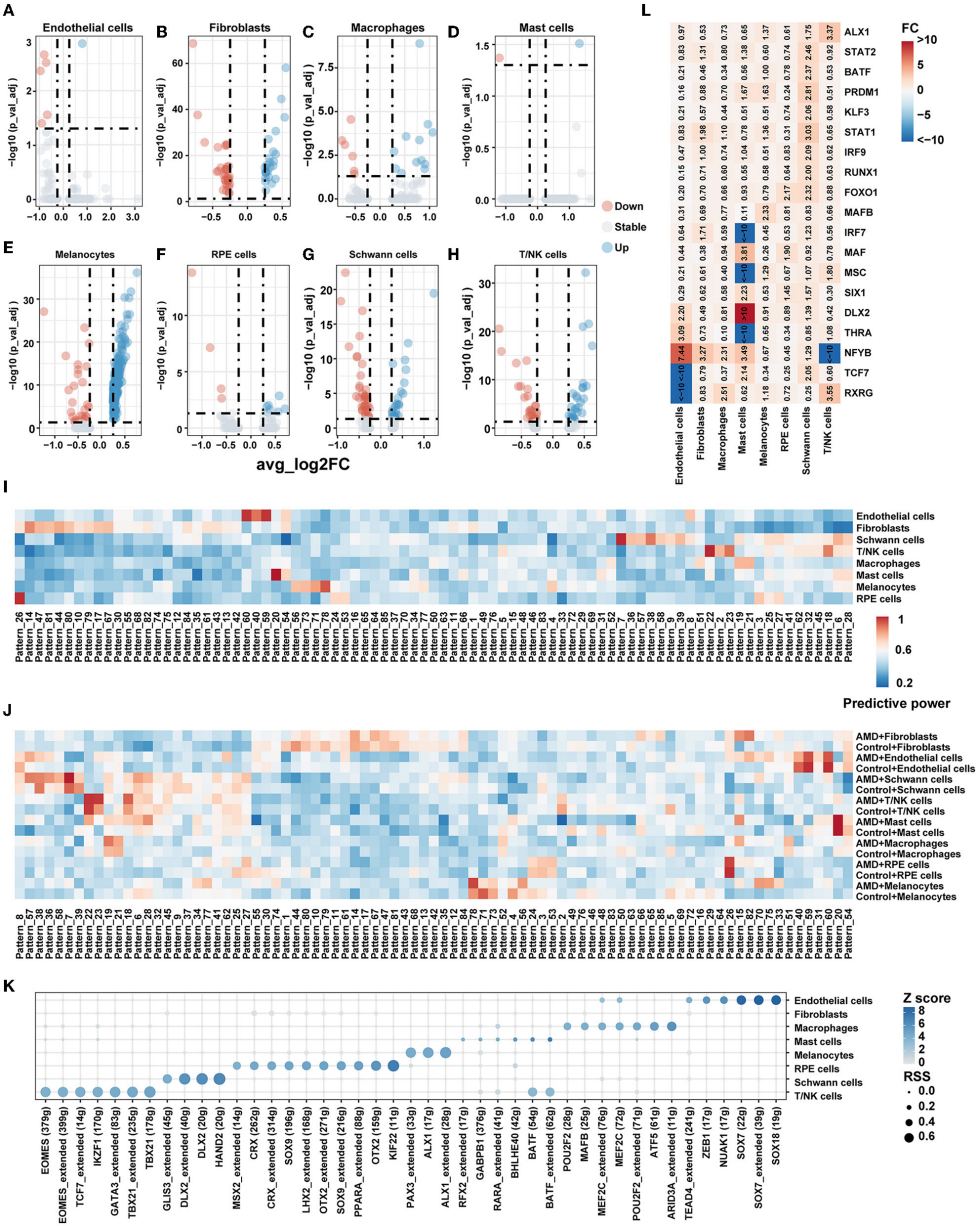
Gene expression pattern. **(A–H)** Volcano plot for the DEG of each cell type between AMD and control. **(I)** Heatmap of predictive power for gene patterns result by scCoGAPS in different celltypes. A higher value indicates greater specificity. **(J)** Heatmap of predictive power for gene patterns result by scCoGAPS in different celltypes between AMD and Control. A higher value indicates greater specificity. **(K)** Transcription factor specificity in each cell type. **(L)** Heatmap of fold change (FC) for TF's RSS between AMD and control.

In order to explore whether changes in cell communication are related to gene expression, the differential expression analysis within each cell population was additionally completed between cells originating from the AMD patients vs. the controls (Figures 3A–H). In total, 419 significantly differentially expressed genes (|avg_log2FC| > 0.25, *p_val_adj* < 0.05) were detected, which are listed in Supplementary Table S5. We performed GO and KEGG pathway enrichment analyses by DAVID (Supplementary Table S6). The annotation reported that the DEGs were consistently enriched in numerous important eye disease-associated functions, containing the retinol metabolic process, apoptotic process, immune response, vasculogenesis, and circadian regulation.

Then we conducted gene mapping to explore whether the genes differentially expressed in each cell type were involved in the significantly changed ligand–receptor pathway between AMD patients and controls. We found that only fibroblasts [AGT pathway (*AGT*) and CXCL (*ACKR3*)], melanocytes [SPP1 pathway (*SPP1*)], Schwann cells [MPZ pathway (*MPZ*), NEGR pathway (*NEGR1*)], and T/NK cells [CXCL pathway (*CXCR4*), ICAM, and JAM pathway (*ITGAL*)] were involved. It suggests that differentially expressed genes may be related to cell communication. However, the differential status of gene expression explained only 12.3% (7/57) of the information flow gain (or loss) between AMD and control samples; additional influencing factors of transcriptional regulation still need to be further explored.

### 3.5. Patterns of gene expression

As we all know, molecules do not act alone but interact with each other and form individual patterns to reflect cell specification and capture key developmental transitions in the biological system. Focusing on finding gene signatures specific to the cell populations across AMD states, we extracted 85 independent patterns of genes across all cells by scCoGAPS. Figure 3I shows the patterns that were maximally correlated with an individual cell type. Pattern_8, Pattern_40, Pattern_54, Pattern_59, and Pattern_60 are closely associated with endothelial cells. The genes in these patterns were enriched in endothelial cell-associated functions, including vasculogenesis, regulation of vascular permeability, branching involved in blood vessel morphogenesis, establishment of endothelial barrier, and endothelial cell proliferation. Pattern_6, Pattern_7, Pattern_9, Pattern_28, Pattern_36, Pattern_38, Pattern_39, Pattern_57, and Pattern_58 are strongly correlated with Schwann cells, mainly involving neuron projection development, neurotransmitter receptor transport, t regulation of cell cycle, Golgi to plasma membrane protein transport, and pathways of neurodegeneration diseases.

We further characterized the patterns between AMD patients and controls. Figure 3J shows some patterns with broadly similar expression patterns highly correlated with individual cell types while displaying discordance between AMD patients and controls. For example, Pattern_6, Pattern_7, Pattern_38, and Pattern_39 are strongly correlated with T/NK cells in both AMD and control, while Pattern_27, Pattern_28, Pattern_36, Pattern_57, and Pattern_58 mark Schwann cells but highlight patients with AMD only. These findings indicated that combinations of gene expression could extract identical corporate models with the ability to effectively distinguish between cell types and disease states of AMD.

### 3.6. Unique transcriptional regulons active across cell types

Cellular identity and how that identity is developed and maintained are critical questions in single-cell experiments. This cell identity major emerges from an underlying gene regulatory network (GRN), in which the coordinated regulation of specific combinations of transcription factors (TFs) drives the expression of their target genes. To better characterize the regulatory networks (regulons) among cell types and disease states, we adopted SCENIC to systematically detect the regulons and assess their cell-type specificity. The unique regulons for each cell type are shown in Figure 3K. Most regulons that only activate in one cell type, such as PAX3 and ALX1 exhibited regulation effects on melanocytes only. While two regulons, *BATF* and *MEF2C*, were active in two of the cell types (*BATF* activates T/NK and mast cells *MEF2C* activates endothelial cells and macrophages). We also found this expression specificity in external dataset 2 (Supplementary Figure S8), where PAX3 and ALX1 were shown to be specifically expressed in Melanocytes.

We found that the main difference in regulon also existed among cell populations of AMD and controls (Figure 3L). Obvious changes in the specific regulator when disease state differences were taken into account. *NFYB* regulon was more activating in endothelial cells (fold change = 7.44), fibroblasts (fold change =3.27), macrophages (fold change =2.31), and mast cells (fold change =3.49), while relatively silent in T/NK cells (fold change < −10) in AMD than controls. *TCF7* regulon was more activating in mast cells (fold change = 2.14) and Schwann cells (fold change = 2.05) but also relatively silent in endothelial cells (fold change < −10). Seven key TF regulons, including *BATF, IRF9, KLF3, PRDM1, RUNX1, STAT1*, and *STAT2*, were shown morn activating merely in Schwann cells. These results were also reproduced in external dataset 2 (Supplementary Figure S9). The above findings indicate that the regulon difference was not merely affected by inter-cell heterogeneity but also by inter-disease states. The regulons would affect disease development through multiple signal paths and could contribute to potential target mining and evaluation.

### 3.7. Cell-specific transcription factor regulatory loops

**Figure 4:**
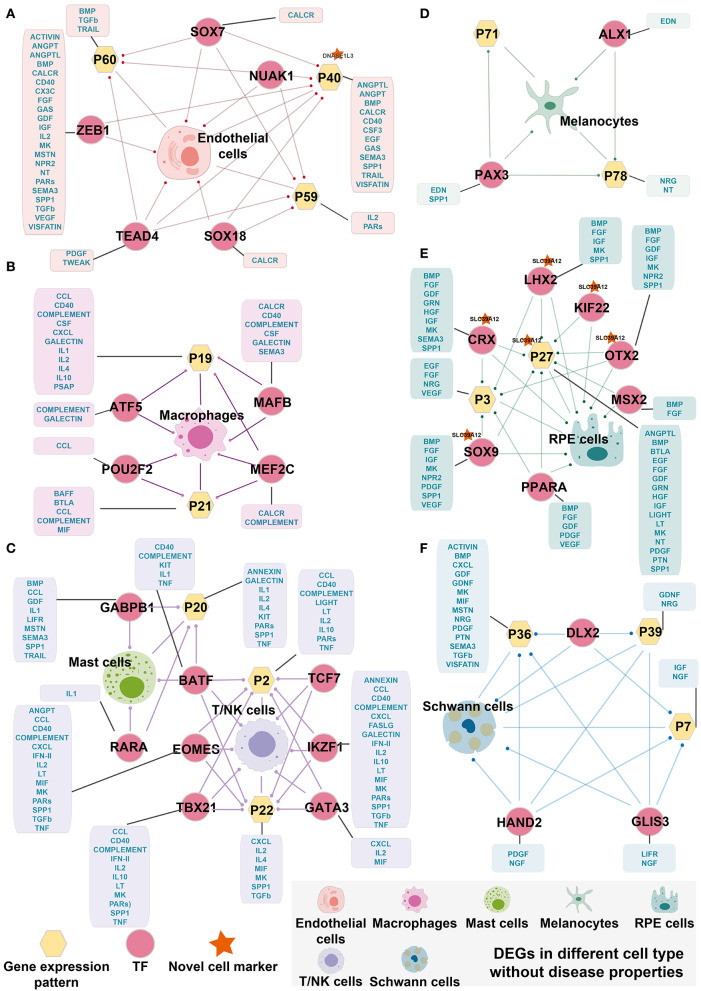
Cell-specific TF regulatory loop without disease properties. **(A)** Endothelial cells-specific TF regulatory loop. **(B)** Macrophages-specific TF regulatory loop. **(C)** Mast cells and T/NK cells-specific TF regulatory loop. **(D)** Melanocytes-specific TF regulatory loop. **(E)** RPE cells-specific TF regulatory loop. **(F)** Schwann cells-specific TF regulatory loop. TFs, gene expression patterns, and DEGSs were marked by pink circles, yellow hexagons, and cells. The yellow hexagons or pink circles, which are marked by a red star indicated that the novel cell marker was contained in the gene expression patterns or the target gene of TF. The yellow hexagon-connected box indicates that the gene expression pattern is directly involved in the signaling pathway of the cell in cell communication, and the box which is connected by pink circles indicates that the TF directly regulates the signaling pathway of cell-in-cell communication. Edges with dots indicate directed targets by TFs.

Although it is widely accepted that gene regulation is variable between AMD patients and controls, the detailed regulation paths and factors that cause the variation are poorly understood. By selecting DEGs, gene expression patterns, and TF regulons mentioned above, we aim to explore whether there are intrinsic conduction relationships and correlations between them. Here, we used the hypergeometric test and found that cell-specific TF can regulate DEGs and related gene expression patterns, forming cell-specific TF regulatory loops (Figure 4). Interestingly, we found that the patterns highly associated with cells tended to be enriched by differentially expressed genes and were associated with signals found in cell communication analysis. The same situation was also discovered in cell-specific TFs. For instance, in Schwann cells, the DEGs, Pattern_7, Pattern_36, and Pattern_39 are enriched by three key TFs' targets, including *DLX2, GLIS3*, and *GLIS3*. These three TF regulons were shown specifically activating only in Schwann cells. Consistently, we also found that these TF and cell-specific gene expression patterns were involved in the multiple signaling pathways for cell communication of endothelial cells, including ACTIVIN, BMP, CXCL, GDF, GDNF, LIFR, MK, MIF, MSTN, NGF, NRG, PDGF, PTN, SEMA3, TGFb, and VISFATINIGF (Figure 4F). Similar results were also seen in endothelial cells, macrophages, mast cells, melanocytes, RPE cells, and T/NK cells (Figures 4A–F). Miraculously, we found that *BATF* was related to both Mast cells and T/NK cells and was enriched with DEGs from both types of cells. Therefore, *BATF* may affect bridging and contribute to the corporation in the expression of regulation of mast cells and T/NK cells.

**Figure 5:**
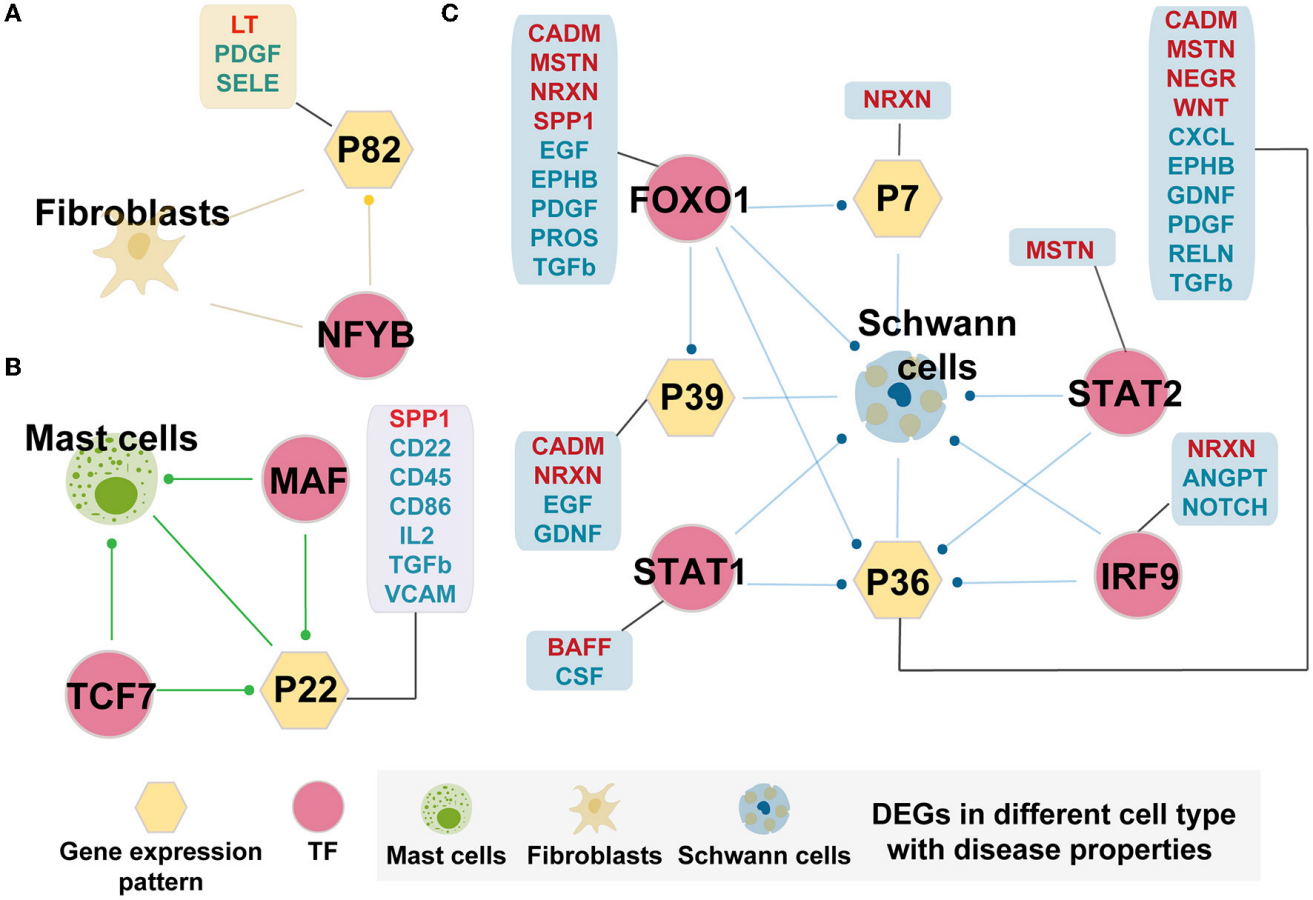
Cell-specific TF regulatory loop with disease properties. **(A)** Fibroblasts-specific TF regulatory loop. **(B)** Mast cells-specific TF regulatory loop. **(C)** Schwann cells-specific TF regulatory loop. TFs, gene expression patterns, and DEGSs were marked by pink circles, yellow hexagons, and cells. The yellow hexagon-connected box indicates that the gene expression pattern is directly involved in the prominently changed pathways of the cell-in-cell communication between AMD and controls (pathway marked in red fonts indicate increased intensity in AMD, and those marked blue fonts indicate decreased intensity in AMD), and the box which connected by pink circles indicates that the TF directly regulates the prominently changed pathways of the cell-in-cell communication between AMD and controls. Edges with dots indicate directed targets by TFs.

We further identified cell-specific TF regulatory loops with disease properties, as shown in Figure 5. There were three specific regulatory loops in the AMD than controls, including fibroblasts, mast cells, and Schwann cells. *NFYB*, which is more activating in fibroblasts in AMD, could target the genes specific to fibroblasts that were differentially expressed, and Pattern_82 which is significantly associated with fibroblasts in AMD (Figure 5A). Meanwhile, Pattern_82 was significantly enriched by DEGs and involved in one enhanced cellular communication signaling pathway (LT pathway) and two diminished pathways (PDGF and SELE pathway) in AMD patients. LT pathway mediates a large variety of inflammatory, immunostimulatory, and antiviral responses. PDGF pathway plays an essential role in the regulation of embryonic development, cell proliferation, and survival. Meanwhile, SELE pathway has roles in cell proliferation, differentiation, motility, trafficking, apoptosis, tissue architecture, and capillary morphogenesis. Therefore, we speculated that *NFYB* could disrupt the normal function of fibroblasts by changing gene expression and further regulating cell communication in the progress of AMD.

Mast cells and Schwann cells also formed the corresponding TF regulatory loops (Figures 5B, C). Interestingly, Schwann cell-related loops could precisely screen the key regulatory molecules involved in disease states. The key role units Pattern_7, Pattern_36, and Pattern_39 were preserved from all the related patterns obtained in the above finding. In addition, the interpretation of the differential change pathways between AMD and control increased from the original two (2/57, 3.5%) pathways to 18 (31.6%, 18/57) pathways (Figure 5C). We also found that they may be directly or indirectly involved in the regulation of the immune and nervous systems (ANGPT, BAFF, CADM, CSF, EGF, EPHB, GFRA1, MSTN, NEGR, NOTCH, PROS, and RELN). Similarly, for mast cells, we detected the essential TFs, *MAF and TCF7*, which can target DEGs and Pattern_22. The corporation of the regulation loop could break the interaction balance between cells by interfering with the functions of CD molecules (CD22, CD45, CD86, and CD96), CXCL, FN1, IL2, TGFb, SPP1, and VCAM pathway. These pathways participate in the mutual recognition of immune cells during the immune response. They also affect the recognition of antigens, activation, proliferation, and differentiation of immune cells, thus affecting the play of immune efficacy and the occurrence of inflammation. These results demonstrated that the extracted key TFs, including *NFYB, MAF, TCF7, FOXO1, IRF9, STAT1*, and *STAT2*, may play a considerable corporation regulation role in the combining of gene patterns and DEGs. The forming cell-specific loops would provide new insight into the potential molecular mechanism and therapeutic targets for AMD.

## 4. Discussion

In this study, we identified the most predominant cell types in RPE-choroid, including endothelial cells, fibroblast, macrophages, mast cells, melanocytes, RPE cells, Schwann cells, and T/NK cells, with their heterogeneities deciphered between AMD patients and controls. In addition, three novel cell markers were discovered, and cell communication, specific signaling pathways, gene expression, and regulators were compared in the case–control group. In addition, computational strategies are developed to construct cell-specific regulatory loops, providing adding evidence for systematic exploring of regulatory modes and molecular mechanisms involved in cell functions in the course of AMD.

We conduct in-depth mining on the single-cell data of transcriptome to identify how different cell types contribute to key biological functions in the progression of AMD. We found that the *ACT* of Schwann cells in AMD patients (1.63) was 2.7 times greater than that of controls (0.60), and that the ACT of Schwann cells in the macula (1.79) was 8.5 times larger than that of peripheral (0.21), implying that most Schwann cells derived from the macula of the donor with neovascular AMD. Voigt et al. also got a similar finding (Voigt et al., 2019). Schwann cells were demonstrated to significantly increase the expression of complement factor D (*CFD*) in AMD patients. *CFD* is a pivotal regulator of the alternative complement pathway and mainly acts early in the alternative complement pathway, which plays an essential role in AMD (Yaspan et al., 2017; Acar et al., 2020). Our report would provide further information on *CFD*, whose potential therapeutic target is the alternative complement pathway in the AMD.

The *ACT* of melanocytes in AMD patients (1.28) was 1.56 times larger than that of controls (0.82). *SPP1* was the most upregulated gene in the AMD-donor melanocytes, which acts as a cytokine involved in enhancing the production of interferon-gamma and interleukin-12 and reducing the production of interleukin-10. It is essential in the pathway that leads to type I immunity. Schlecht et al. identified *SPP1* as one of the most highly expressed genes in retinal microglia in the course of CNV formation and investigated the role of *SPP1* in CNV formation by local intraocular application of an antibody directed against *SPP1*. These results underline the importance of *SPP1* in the formation of CNV, thus paving the way for new interventions by modulating the SPP1 pathway (Schlecht et al., 2020).

In the survey of the variant cell–cell communications in AMD and controls, numerous interactional pairs were identified between cell types, indicating that these cells participate in the maintenance of RPE/choroid homeostasis. However, a large number of ligand–receptor pairs related to multiple vital signaling pathways were erased in AMD patients. We spotted that *CD70*-*CD27* (CD70 pathway), *CX3CL1*-*CX3CR1* (CX3C pathway), *SELE*-*CD44* (SELE pathway), and *TNF*-*TNFRSF1A* (TNF pathway), which have been reported to play critical roles in inflammatory and immune response, were erased in AMD patients, implying their impact on the inflammation and immune response in AMD. In addition, *GH1*-*GHR* (GH pathway), *GP1BA*-(*ITGAM*+*ITGB2*) (GP1BA pathway), *MSTN*-(*TGFBR1*+*ACVR2B*) (MSTN pathway), and *WNT3A*-(*FZD1*+*LRP5*) (WNT pathway) were disappeared in controls, indicating that its deficiency might be one of the underlying causes for the onset or deterioration of AMD.

We used the scCoGAPS algorithm to identify gene patterns specific to the cell type without and with disease state properties. In investigating gene expression patterns with disease states properties, some patterns were specific to cell types that conserved across disease states (AMD or controls), but some were not. Looking at some of these patterns, we understood how the major cells in AMD patients differ from controls. Beyond that, we also investigated cell-specific transcription factor regulons and compared their activity in AMD patients with controls. Integrated DEGs, gene expression patterns, and transcriptional regulons results, we found that the perturbation of 7 transcription factors, including *NFYB, MAF, TCF7, FOXO1, IRF9, STAT1*, and *STAT2* between AMD patients and controls, might play momentous parts in the development of AMD. Among them, *FOXO1, IRF9, STAT1*, and *STAT2* can affect the pathogenesis of AMD at both the transcriptional expression and cell communication levels. Although there is no evidence that they are directly related to AMD, studies link them to eye development. For example, Anand et al. showed that *MAF* had a vital function in eye development and was related to cataracts, among other defects, in human patients (Anand et al., 2018). Cui et al. found that *TCF7* mediates the function of canonical WNT signaling, plays a pivotal part in the control of ESC-RPC differentiation and proliferation, and is closely associated with the mouse retina's early development (Wang et al., 2019). *FOXO1* regulates apoptosis, autophagy, anti-oxidative stress, cell cycle arrest, metabolism, and other physiological and pathophysiological processes. MiR-27a-FOXO1 axis plays a prominent role in modulating reactive oxygen species-mediated retinal injury and RPE cell death, which has long been considered a contributor to the onset of AMD (Ren et al., 2021). It is also known that *STAT1*-deficient mice are highly susceptible to autoimmune disorders, and given that AMD may be considered an autoimmune disease, preserving *STAT1* activation may be necessary for mitigating AMD progression (Jiang et al., 2013). All of these findings suggest that key TFs may be of the essence to the pathogenesis of AMD. However, their direct association with AMD requires further investigation of the integers, which can be used as potential targets for the research and treatment of AMD.

Collectively, we observed altered cell–cell communication in patients with AMD, which might result in the disorder of normal cellular function. Our study delivered a comprehensive single-cell transcriptomic in-depth analysis framework for deciphering gene expression landscapes of heterogeneous cell types in AMD patients and controls.

## Data availability statement

The original contributions presented in the study are included in the article/Supplementary material, further inquiries can be directed to the corresponding authors.

## Ethics statement

Ethical review and approval was not required for the study on human participants in accordance with the local legislation and institutional requirements. Written informed consent for participation was not required for this study in accordance with the national legislation and the institutional requirements.

## Author contributions

LX, HW, and JS: conception and design of the study and critical revision of the manuscript. WW and PL: statistical analysis and interpretation of data. SW, GZ, XL, and PL: drawing the illustrations. CC and YZ: administrative, technical, and material support. HW and WW: drafting of the manuscript. All authors read and approved the final manuscript.

## Supplementary material

Supplementary Figure S1 ElbowPlot. Horizontal axis shows the number of principal components, and the vertical axis shows the standard deviation. The smaller the standard deviation, the more representative the principal component.

Supplementary Figure S2 Three-dimensional spatial distribution of principal components 1, 2, 3 of cell types. **(A)** Three-dimensional spatial distribution of principal components 1, 2, 3 of cell types in this study. **(B)** Three-dimensional spatial distribution of principal components 1, 2, 3 of cell types in data source's study. Different colors represent different cell types.

Supplementary Figure S3 Repetition was performed in the external validation set. **(A)** Expression of the top five genes of each cluster in external dataset 1. **(B)** The expression of the novel cell marker in external dataset 1.

Supplementary Figure S4 Interaction strength of each cell with other cells. A thicker line indicates a stronger interaction strength between cells.

Supplementary Figure S5 Information flow in EAMD and control in external dataset 2. “Turn off” represents the closed path in AMD, “Decrease” represents the reduced path in AMD, “Increase” represents the increased path in AMD, and “Turn on” represents the open path only in AMD.

Supplementary Figure S6 Overall signal patterns in control, early AMD (EAMD), and AMD. The bar graph above shows the total strength of each signal of all cells, and the bar graph on the right shows the total strength of each cell of all signals. The darker the color of the heat map, the stronger the signal strength.

Supplementary Figure S7 Cell communication patterns. **(A)** Inferred outgoing communication patterns of secreting cells in AMD show the correspondence between the inferred latent patterns and cell groups, as well as signaling pathways. The thickness of the flow indicates the contribution of the cell group or signaling pathway to each latent pattern. **(B)** The inferred incoming communication patterns of target cells in AMD. **(C)** The inferred outgoing communication patterns of secreting cells in control. **(D)** The inferred incoming communication patterns of target cells in control.

Supplementary Figure S8 Transcription factor specificity in each cell type in external dataset 2.

Supplementary Figure S9 Heatmap of fold change (FC) for TF between AMD and control in external dataset 2.

Supplementary Table S1 Information of the three donors in external dataset 2.

Supplementary Table S2 Information about the three donors included in the single-cell transcriptomic datasets.

Supplementary Table S3 Summary of the extracted clusters in the single-cell transcriptomic datasets.

Supplementary Table S4 Summary of interaction strength.

Supplementary Table S5 A total of 419 significantly differentially expressed genes.

Supplementary Table S6 GO and KEGG enrichment results of differentially expressed genes in different cell types.
