# Supplementary figures and images for: In-depth mining of single-cell transcriptome reveals the key immune-regulated loops in age-related macular degeneration
Source: Front Mol Neurosci. 2023 May 19;16:1173123. doi: 10.3389/fnmol.2023.1173123 (PMC10235539; doi:10.3389/fnmol.2023.1173123)

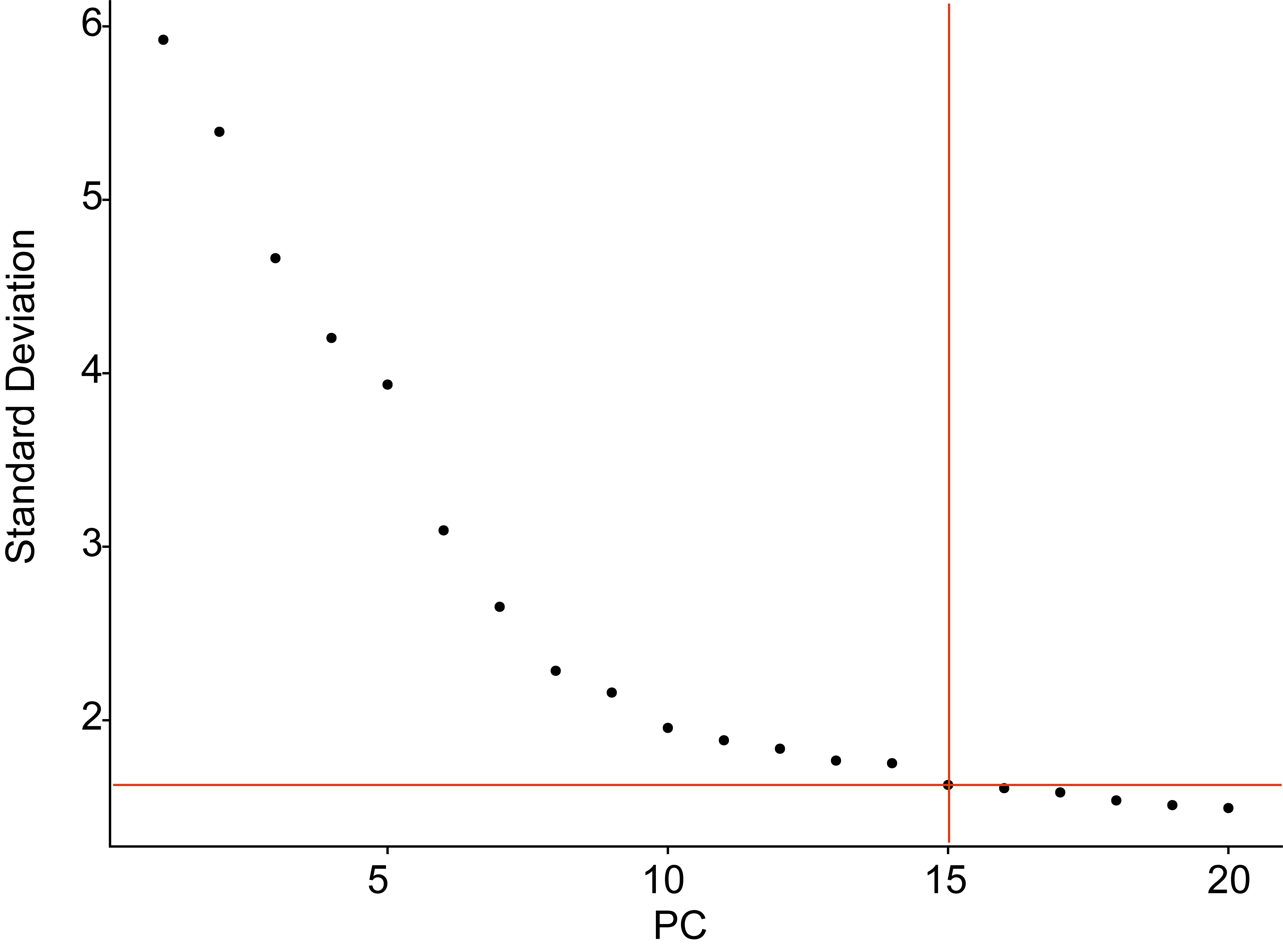

Supplement: Supplementary Figure S1 — ElbowPlot. Horizontal axis shows the number of principal components, and the vertical axis shows the standard deviation. The smaller the standard deviation, the more representative the principal component. [file Image_1.TIF]

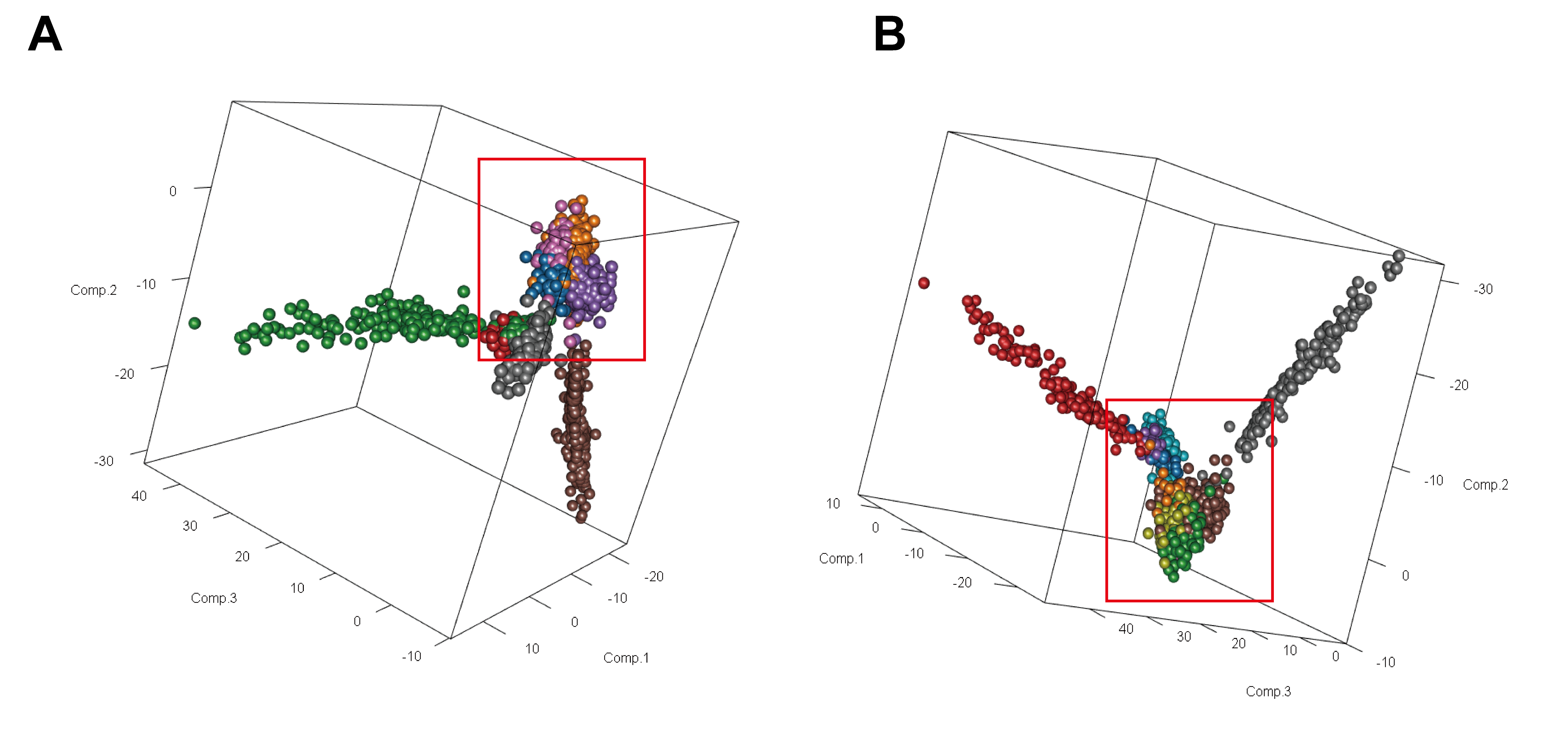

Supplement: Supplementary Figure S2 — Three-dimensional spatial distribution of principal components 1, 2, 3 of cell types. (A) Three-dimensional spatial distribution of principal components 1, 2, 3 of cell types in this study. (B) Three-dimensional spatial distribution of principal components 1, 2, 3 of cell types in data source's study. Different colors represent different cell types. [file Image_2.TIF]

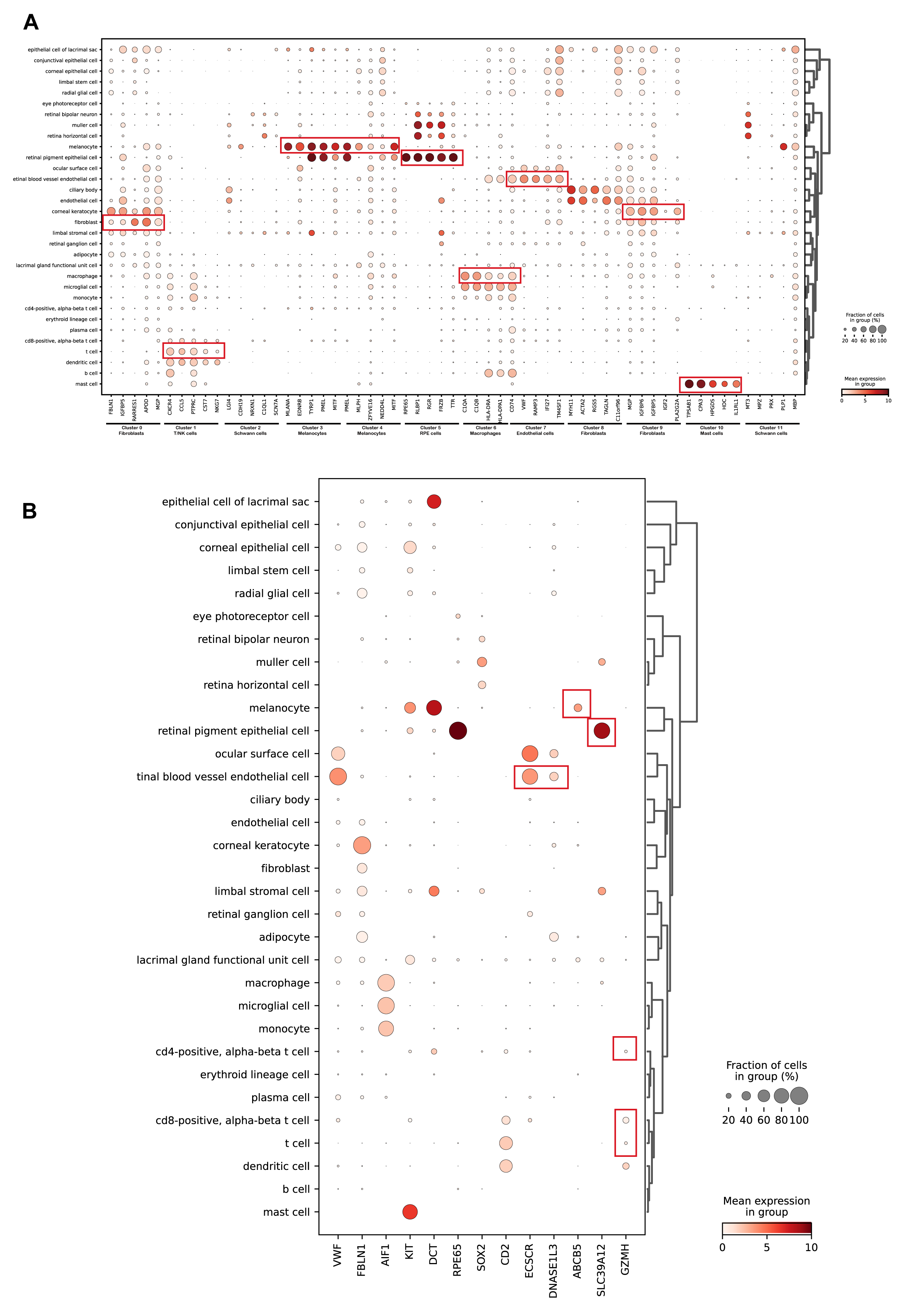

Supplement: Supplementary Figure S3 — Repetition was performed in the external validation set. (A) Expression of the top five genes of each cluster in external dataset 1. (B) The expression of the novel cell marker in external dataset 1. [file Image_3.TIF]

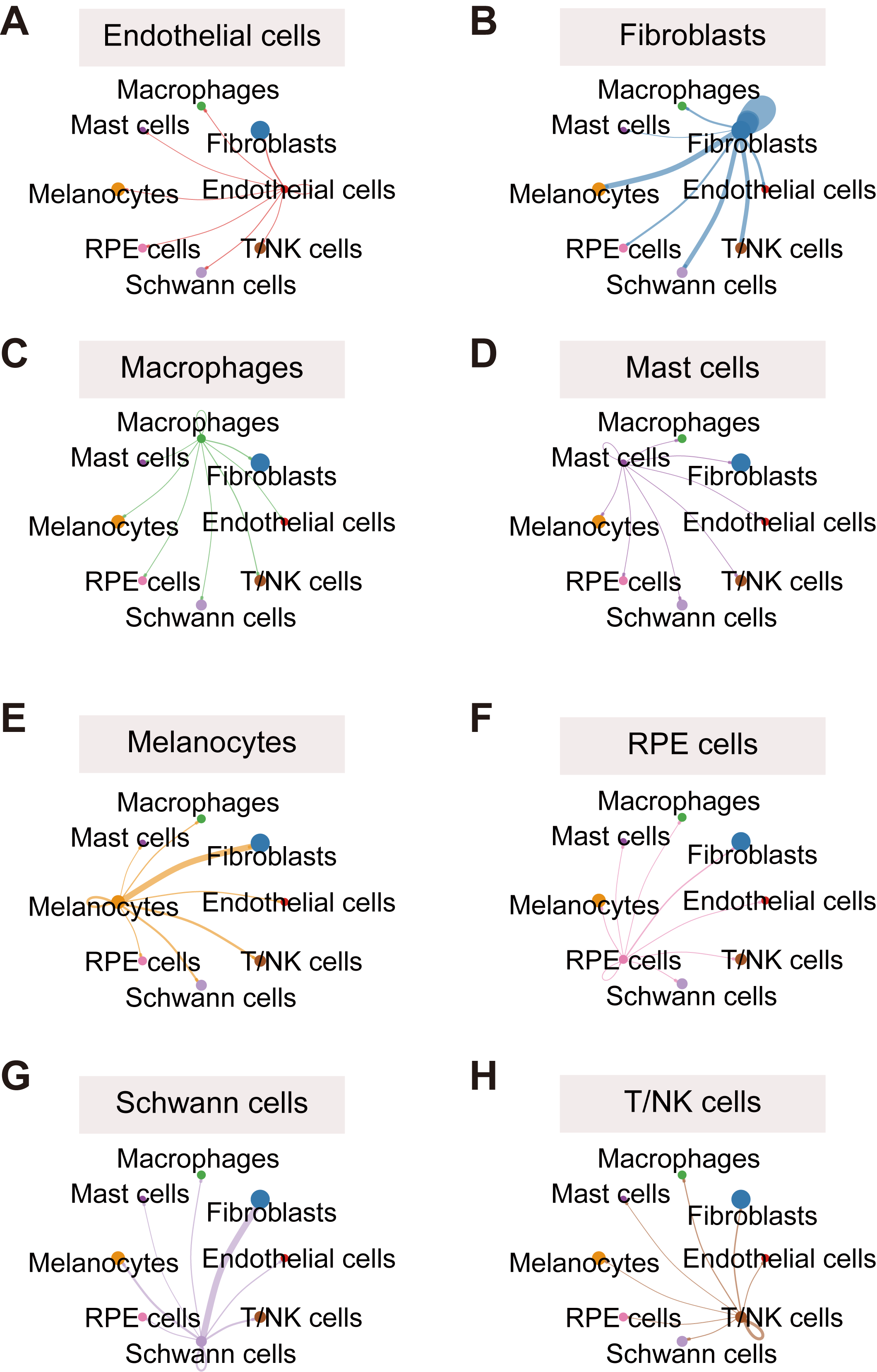

Supplement: Supplementary Figure S4 — Interaction strength of each cell with other cells. A thicker line indicates a stronger interaction strength between cells. [file Image_4.TIF]

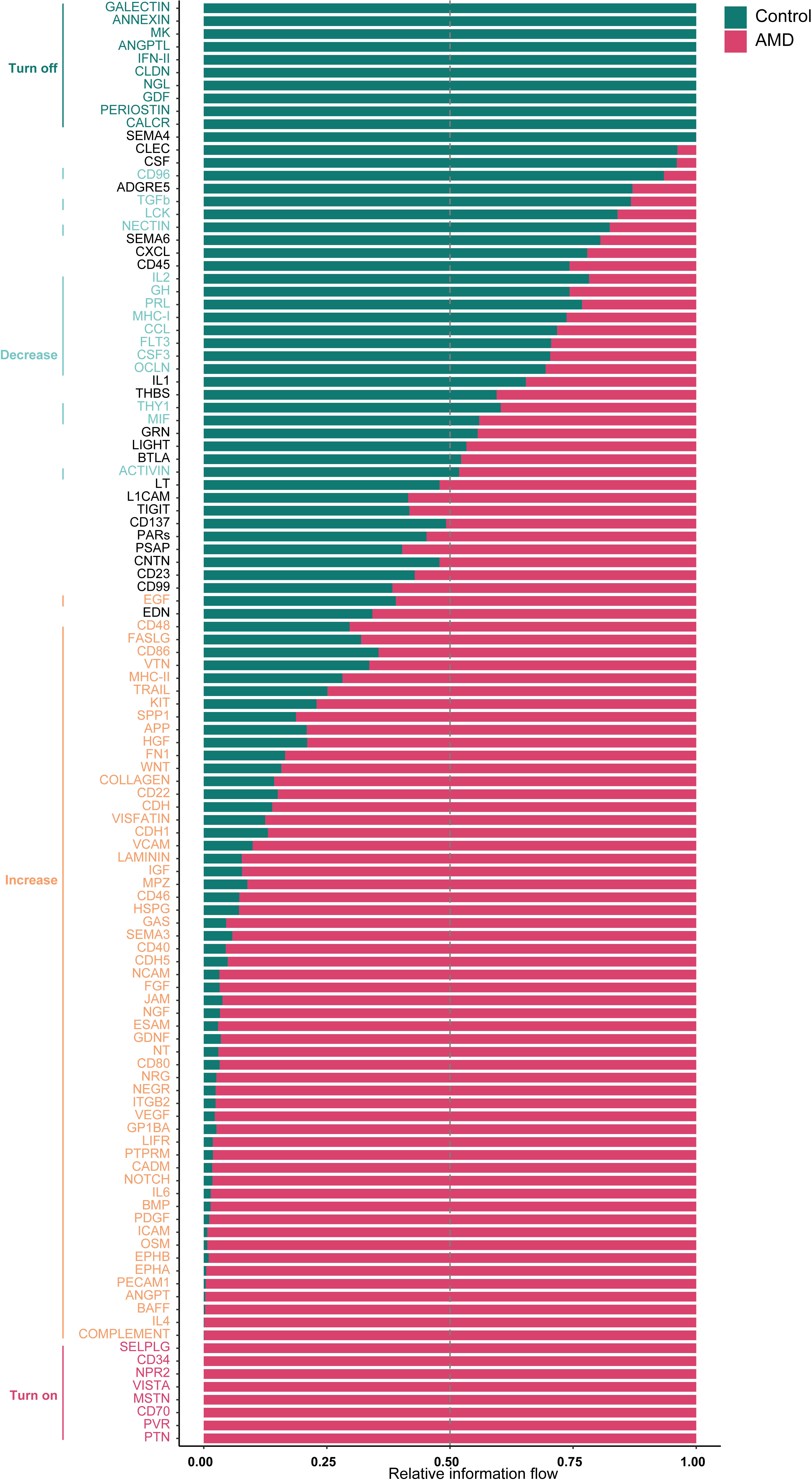

Supplement: Supplementary Figure S5 — Information flow in EAMD and control in external dataset 2. “Turn off” represents the closed path in AMD, “Decrease” represents the reduced path in AMD, “Increase” represents the increased path in AMD, and “Turn on” represents the open path only in AMD. [file Image_5.TIF]

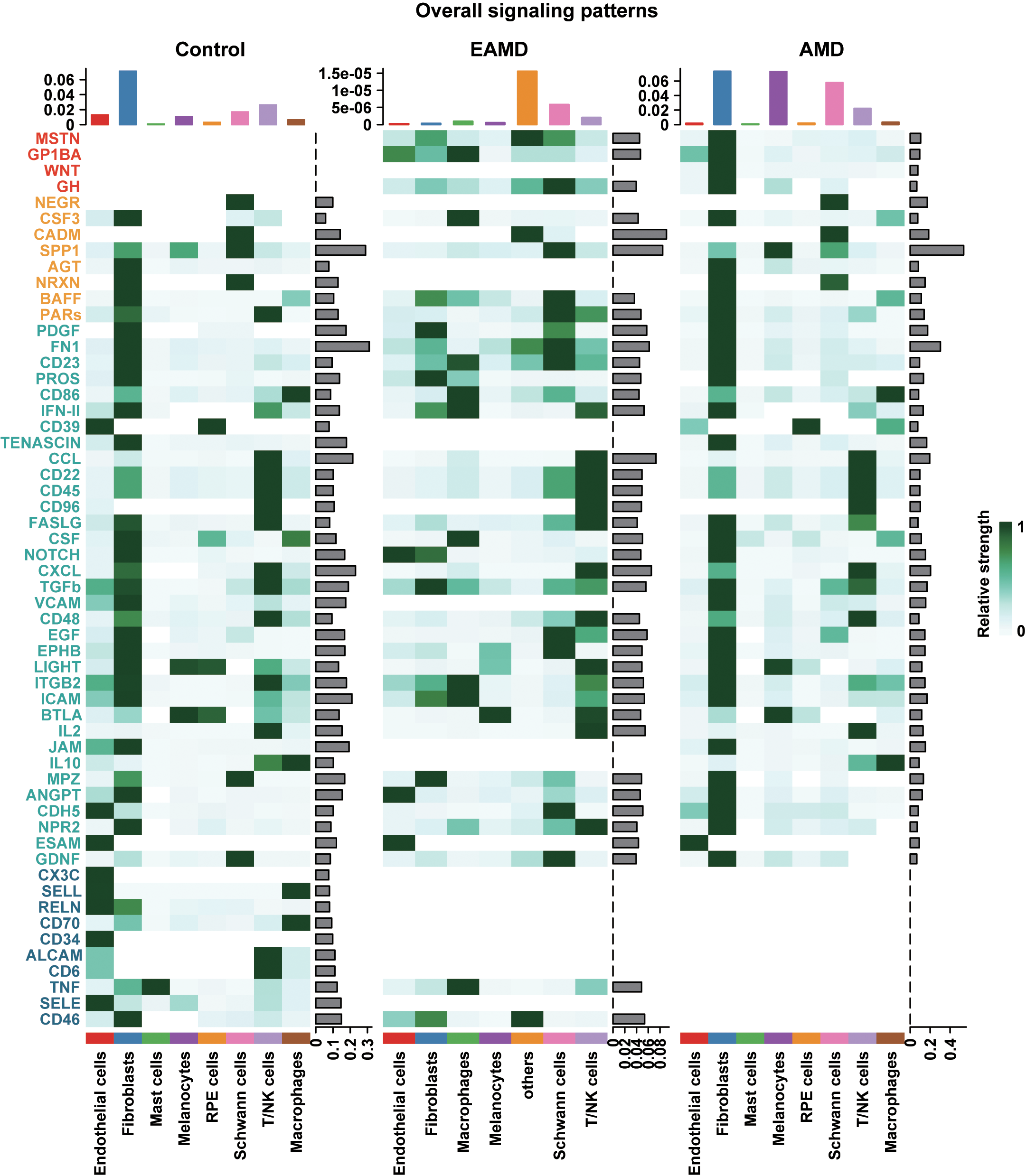

Supplement: Supplementary Figure S6 — Overall signal patterns in control, early AMD (EAMD), and AMD. The bar graph above shows the total strength of each signal of all cells, and the bar graph on the right shows the total strength of each cell of all signals. The darker the color of the heat map, the stronger the signal strength. [file Image_6.TIF]

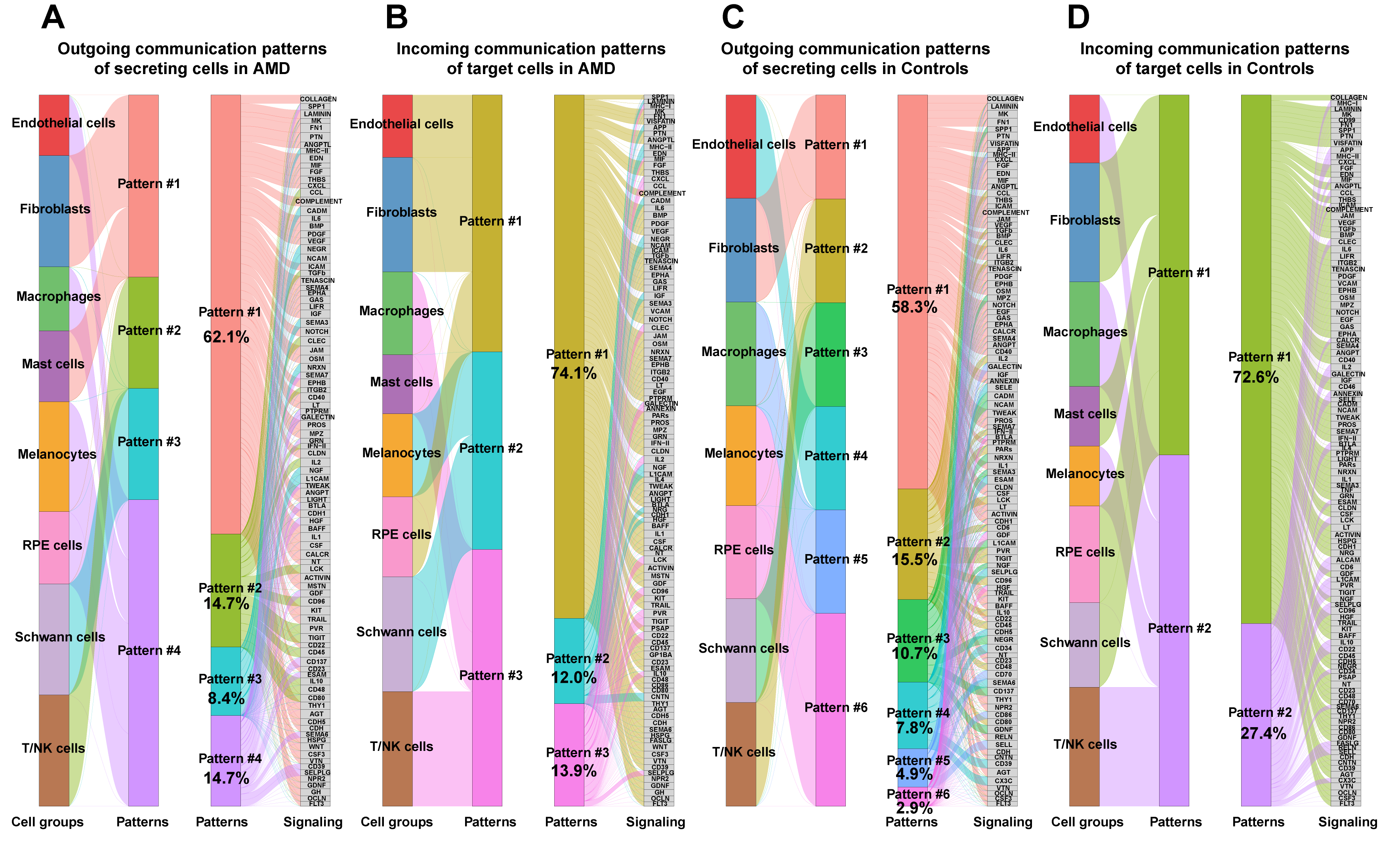

Supplement: Supplementary Figure S7 — Cell communication patterns. (A) Inferred outgoing communication patterns of secreting cells in AMD show the correspondence between the inferred latent patterns and cell groups, as well as signaling pathways. The thickness of the flow indicates the contribution of the cell group or signaling pathway to each latent pattern. (B) The inferred incoming communication patterns of target cells in AMD. (C) The inferred outgoing communication patterns of secreting cells in control. (D) The inferred incoming communication patterns of target cells in control. [file Image_7.TIF]

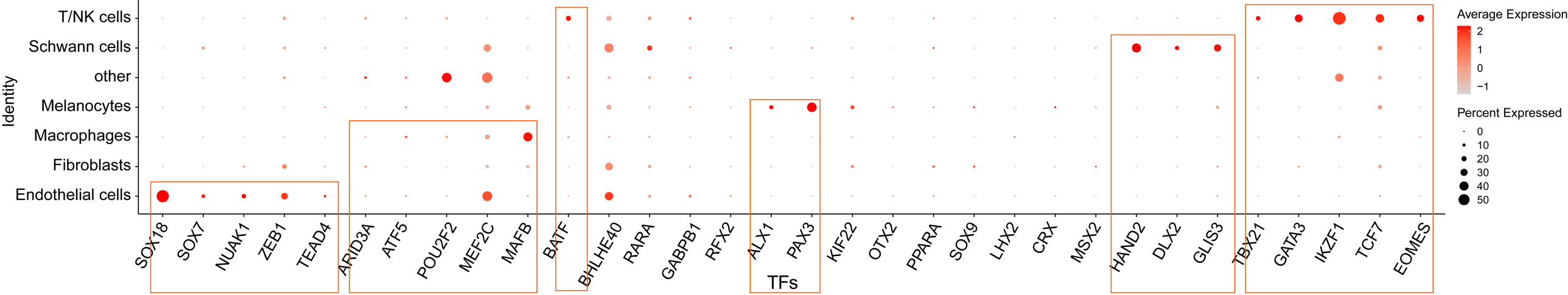

Supplement: Supplementary Figure S8 — Transcription factor specificity in each cell type in external dataset 2. [file Image_8.TIF]

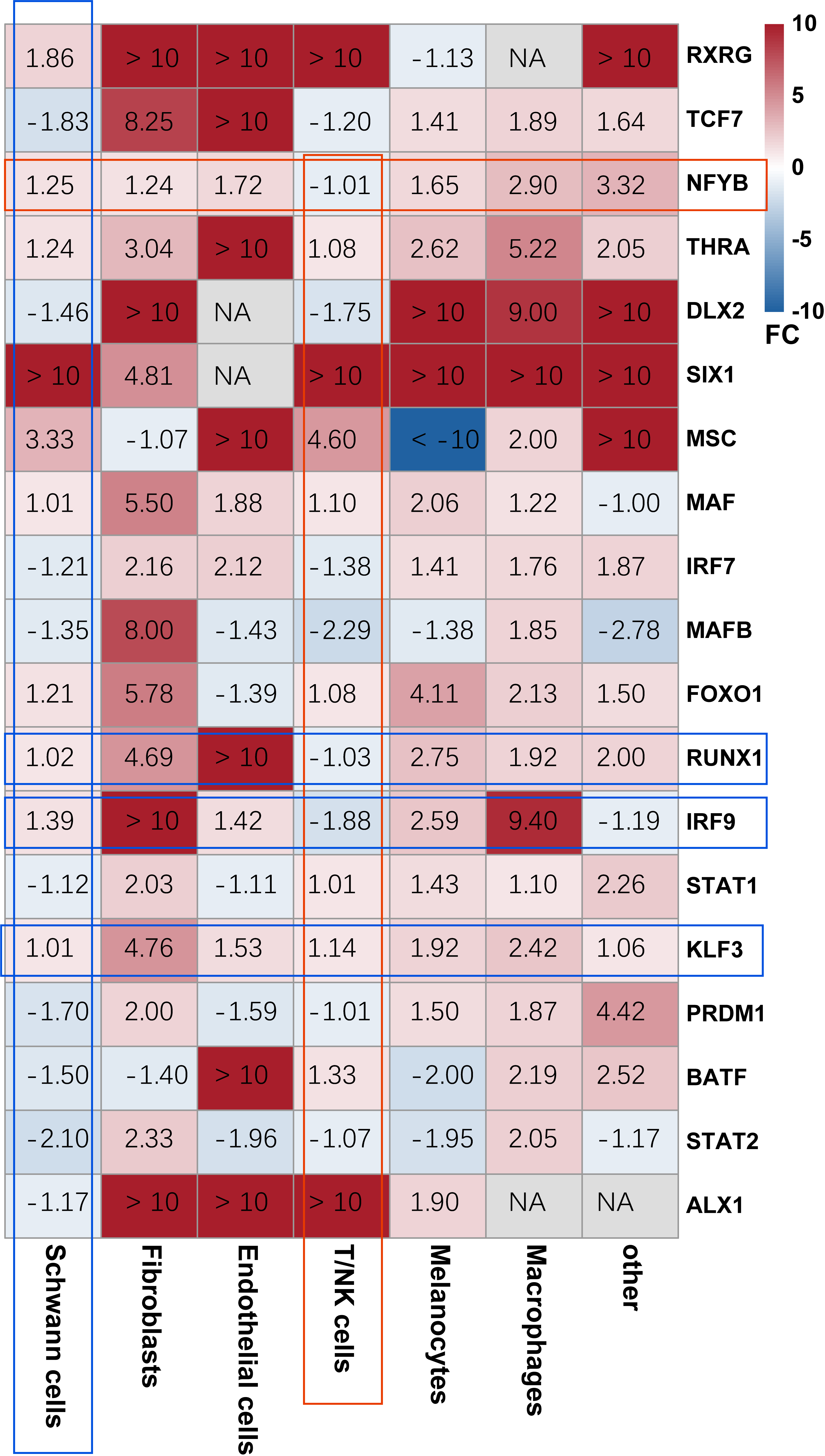

Supplement: Supplementary Figure S9 — Heatmap of fold change (FC) for TF between AMD and control in external dataset 2. [file Image_9.TIF]
