# Supplementary material for: In-depth mining of single-cell transcriptome reveals the key immune-regulated loops in age-related macular degeneration
Source: Front Mol Neurosci. 2023 May 19;16:1173123. doi: 10.3389/fnmol.2023.1173123 (PMC10235539; doi:10.3389/fnmol.2023.1173123)
Supplement: Supplementary Table S1 — Information of the three donors in external dataset 2. [file Table_1.DOCX]

**Supplementary Table S1: Information of the three donors in external dataset 2.**

| **Donor** | **Sample ID** | **Group** | **Location** | **NO. of Cells** |
| --- | --- | --- | --- | --- |
| 715 | GSM5676873 | Early AMD | Macula | 6,518 |
| 715 | GSM5676874 | Early AMD | Macula | 13,591 |
| 715 | GSM5676875 | Early AMD | Periphery | 10,347 |
| 715 | GSM5676876 | Early AMD | Periphery | 11,807 |
| 828 | GSM5676877 | Normal | Macula | 3,326 |
| 828 | GSM5676878 | Normal | Macula | 12,580 |
| 828 | GSM5676879 | Normal | Periphery | 5,995 |
| 828 | GSM5676880 | Normal | Periphery | 10,287 |
| 902 | GSM5676881 | Early AMD | Macula | 3,430 |
| 902 | GSM5676882 | Early AMD | Macula | 13,367 |
| 902 | GSM5676883 | Early AMD | Periphery | 4,095 |
| 902 | GSM5676884 | Early AMD | Periphery | 10,237 |
| All |  |  |  | 105,580 |

**Supplementary Table S2: Information of the three donors included** **in the single-cell transcriptomic datasets.**

| **Donor** | **Sample ID** | **Group** | **Location** | **NO. of Cells** |
| --- | --- | --- | --- | --- |
| Donor 1 | GSM4037981 | Control 1 | Macula | 544 |
| Donor 2 | GSM4037982 | Control 2 | Macula | 567 |
| Donor 3 | GSM4037983 | AMD | Macula | 1,056 |
| Donor 1 | GSM4037984 | Control 1 | Periphery | 851 |
| Donor 2 | GSM4037985 | Control 2 | Periphery | 672 |
| Donor 3 | GSM4037986 | AMD | Periphery | 645 |
| All |  |  |  | 4,335 |

**Supplementary Table S3: A summary of the extracted clusters in the single-cell transcriptomic datasets.**

| **Cluster** | **Cell type** | **Macula** | | |  | **Periphery** | | | **All** |
| --- | --- | --- | --- | --- | --- | --- | --- | --- | --- |
|  |  | **Donor 1** | **Donor 2** | **Donor 3** |  | **Donor 1** | **Donor 2** | **Donor 3** |  |
| C0 | Fibroblasts | 105 | 175 | 249 |  | 255 | 297 | 164 | 1,245 |
| C1 | T/NK cells | 178 | 93 | 119 |  | 41 | 97 | 85 | 613 |
| C2 | Schwann cells | 63 | 82 | 314 |  | 12 | 5 | 12 | 488 |
| C3 | Melanocytes | 16 | 3 | 158 |  | 99 | 45 | 165 | 486 |
| C4 | Melanocytes | 32 | 93 | 63 |  | 111 | 31 | 51 | 381 |
| C5 | RPE cells | 4 | 24 | 27 |  | 120 | 43 | 67 | 285 |
| C6 | Macrophages | 55 | 64 | 35 |  | 43 | 42 | 32 | 271 |
| C7 | Endothelial cells | 31 | 3 | 13 |  | 71 | 49 | 14 | 181 |
| C8 | Fibroblasts | 38 | 13 | 49 |  | 10 | 29 | 17 | 156 |
| C9 | Fibroblasts | 19 | 16 | 17 |  | 26 | 30 | 19 | 127 |
| C10 | Mast cells | 0 | 1 | 1 |  | 37 | 4 | 19 | 62 |
| C11 | Schwann cells | 3 | 0 | 11 |  | 26 | 0 | 0 | 40 |
| All |  | 544 | 567 | 1,056 |  | 851 | 672 | 645 | 4,335 |

**Supplementary Table S4:** **Summary of Interaction strength.**

| Cell type | Interaction strength in AMD patient | | | Interaction strength in Healthy controls | | |
| --- | --- | --- | --- | --- | --- | --- |
|  | All | Sender | Receptor | All | Sender | Receptor |
| Endothelial cells | 0.012 | 0.004 | 0.008 | 0.071 | 0.029 | 0.042 |
| Fibroblasts | 0.508 | 0.312 | 0.196 | 0.529 | 0.327 | 0.202 |
| Macrophages | 0.025 | 0.009 | 0.016 | 0.049 | 0.017 | 0.032 |
| Mast cells | 0.004 | 0.001 | 0.003 | 0.005 | 0.001 | 0.004 |
| Melanocytes | **0.209** | 0.099 | 0.111 | 0.049 | 0.009 | 0.040 |
| RPE cells | 0.017 | 0.004 | 0.013 | 0.028 | 0.008 | 0.020 |
| Schwann cells | **0.230** | 0.102 | 0.128 | 0.066 | 0.036 | 0.031 |
| T/NK cells | 0.113 | 0.029 | 0.085 | 0.144 | 0.043 | 0.101 |
